# Basic life support competency among healthcare professionals in Ethiopia: a systematic review and meta-analysis, 2025

**DOI:** 10.1016/j.resplu.2025.101098

**Published:** 2025-09-11

**Authors:** Gebremeskel Kibret Abebe, Addis Wondmagegn Alamaw, Biruk Beletew Abate, Befkad Derese Tilahun, Alemu Birara Zemariam, Tesfaye Engdaw Habtie, Fekadu Takele Wolie

**Affiliations:** a Department of Emergency and Critical Care Nursing, School of Nursing, College of Health Sciences, Woldia University, Woldia, Ethiopia; b Department of Emergency and Critical Care Nursing, School of Nursing, College of Medicine and Health Sciences, Woldia University, Woldia, Ethiopia; c School of Population Health, Curtin University, Perth, WA, Australia; d Department of Nursing, College of Health Sciences, Woldia University, Woldia, Ethiopia; e Department of Pediatrics and Child Health Nursing, School of Nursing, College of Health Sciences, Woldia University, Woldia, Ethiopia; f Department of Adult Health Nursing, School of Nursing, College of Health Sciences, Woldia University, Woldia, Ethiopia; g Department of Emergency and Critical Care Nursing, Metema General Hospital, Amhara Health Bureau, Ethiopia

**Keywords:** Basic life support, Knowledge, Attitude, Healthcare professionals, Ethiopia

## Abstract

**Background:**

Sudden cardiac arrest is a major global health problem, accounting for nearly half of all cardiovascular deaths. Its burden is particularly high in low-income regions, partly due to limited healthcare facilities. Early Cardiopulmonary resuscitation (CPR) can increase survival rates to 50–70%. However, only a small proportion of cardiac arrest victims receive timely, adequate life-saving cardiopulmonary resuscitation, highlighting an urgent need for improvement.

**Objectives:**

To conduct a systematic review and meta-analysis of knowledge, attitude, and associated factors on basic life support among healthcare professionals in Ethiopia.

**Methods:**

A systematic review and meta-analysis were conducted by searching several databases, including PubMed, Medline, the Cochrane Database of Systematic Reviews, UpToDate, EMBASE, and the National Institute for Health and Care Excellence (NICE). Data on knowledge, attitudes, and associated factors related to basic life support among healthcare professionals were extracted. The pooled estimate of knowledge and attitude levels was calculated using a random-effects model. The Joanna Briggs Institute’s (JBI) critical appraisal checklist was used to assess the quality of the included studies.

**Results:**

The final analysis included 12 studies with a total of 3045 participants. The pooled prevalence of good knowledge of basic life support among healthcare professionals in Ethiopia was 42.87 % (95 % CI: 29.15–56.59 %; I^2^ = 98.86 %; p < 0.001). The pooled prevalence of favorable attitudes towards basic life support was 71.46 % (95 % CI: 69.89–73.04 %; I^2^ = 98.80 %; p < 0.0001). Gender (being male; AOR = 0.31; 95 % CI: 0.07–0.56) was negativity associated with knowledge, while previous exposure to basic life support (AOR = 1.74; 95 % CI: 1.38–2.11) was positively associated. Assessment of publication bias revealed no evidences bias.

**Conclusion:**

The overall prevalence good knowledge among healthcare professionals in Ethiopia remains below the Minimum standards, while their attitude towards basic life support was suboptimal. Male healthcare professionals less likely to have adequate knowledge on basic life support, where as those with pervious exposure to basic life support demonstrate good knowledge.

## Background

Basic life support (BLS) refers to the preservation or restoration of life through the maintenance of airways, breathing, and circulation, along with essential emergency care services.^1^ It represents the initial level of care for patients with life-threatening illnesses or injuries, enabling prompt recognition and emergency support for ventilation and circulation in case of respiratory or cardiac arrest.^2^, ^3^, ^4^

Sudden cardiac arrest is a major global health problem, accounting for nearly half of all cardiovascular-related deaths.^5^ Its burden is high in low-income regions, partly due to limited healthcare facilities.^6^, ^7^ Cardiac arrest, whether occurring in and out of the hospital, remains a significant public health concern and a leading cause of mortality worldwide.^8^ It can result from various factors, including traumatic injuries, cardiac diseases, and stroke.^9^ In hospitalized patients, cardiac arrest can occur unexpectedly and is associated with a high mortality rate if not recognized and managed promptly.^10^

Effective survival and recovery of adult cardiac arrest patients require a well-coordinated system to ensure the best possible outcomes. This includes rapid recognition, prompt cardiopulmonary resuscitation (CPR), defibrillation of shockable rhythms, and post-ROSC supportive care, along with treatment of causes.^11^ Survival can be increased up to threefold when cardiac arrests are managed by individuals trained to provide immediate BLS.^12^, ^13^ Moreover, effective management of the chain of survival may increase survival by 50 % and early defibrillation within three minutes can improve survival rates up to 70 %.^14^, ^15^

However, only a small proportion of cardiac arrest victims receive adequate life-saving cardiopulmonary resuscitation (CPR), highlighting the need for improvement.^12^, ^16^ Only 40 % of out-of-hospital cardiac arrests receive assistance from untrained bystanders.^13^ The knowledge and skills of healthcare providers are critical in determining successful patient outcomes and survival following cardiac arrest.^17^, ^18^, ^19^ All healthcare professionals are expected to be competent in basic life support. Adequate knowledge, skill, and attitude regarding maneuvers and techniques can prevent irreversible organ damage, increase the survival chain of cardiac arrest victims, and reduce post-arrest morbidity.^20^, ^21^, ^22^, ^23^

However, gaps in knowledge and skills among healthcare professionals regarding BLS and cardiac arrest management are a global concern.^19^ In Ethiopia, several studies have assessed knowledge, attitude, and associated factors towards BLS. These studies report highly variable findings, with the prevalence of adequate knowledge ranging from 15 % to 92.7 %, and a favorable attitude ranging from 35.8 % to 94 %. Given these inconsistent results, this systematic review and meta-analysis aimed to provide a pooled estimate of knowledge, attitude, and its associated factors related to BLS among healthcare professionals in Ethiopia, thereby offering more comprehensive and reliable findings to inform stakeholders.

## Methods and materials

### Data sources and searching strategies

All methods employed in this meta-analysis adhered to the PRISMA guidelines for observational research (Supplemental Table 1).^24^ A comprehensive literature search was conducted from May 2014 to June 2025 by an author. The search included PubMed, Medline, the Cochrane Database of Systematic Reviews, Up-to-date, EMBASE, and NICE (National Institute for Health and Care Excellence) to identify relevant studies. Ongoing systematic reviews were identified through PROSPERO registry.

Medical Subject Headings (MeSH) and text terms such as BLS, CPR, basic life support, cardiopulmonary resuscitation, knowledge and/or attitude, and related factors were searched, either separately or in combination, in PubMed. Furthermore, hand searches of reference lists, modified searches for the other databases, and forward citation searches utilizing Web of Science and Google Scholar were also carried out. The search also encompasses unpublished and grey literatures.

The following search map was used: knowledge AND/OR attitude (BLS OR basic life support OR CPR OR cardiopulmonary resuscitation) AND (“healthcare professionals”) AND (Ethiopia).

These terms were further paired with the names of Ethiopian regions, including Addis Ababa, Amhara, South Ethiopia, Tigray, Somalia, and Oromia (Supplementary Table 1).

Using those key terms, we employed the Boolean operators “OR” to join important terms or phrases within the same concept, “AND” (to join key terms or phrases between two concepts), and “NOT” to filter out. Moreover, we used truncation (*), adjacency searching (ADJn), and wildcard symbols to find variations in spelling and variant word endings on the Ovid databases. Also, we applied relevant limits (filters), such as a limit to human studies only.

### Study selection and screening

In this systematic review and meta-analysis, we included studies that assessed knowledge, attitude, and associated factors related to basic life support (BLS) among healthcare professionals in Ethiopia, regardless of study design. Studies were excluded if they did not evaluate the knowledge, attitude, or factors related to Basic life support, did not include health professionals, were not conducted in Ethiopia, or lacked sufficient data for analysis. Studies published only in English and as full manuscripts were included in the analysis. Although the inclusion criteria permitted various study design, all eligible studies identified were cross-sectional study.

All retrieved records were exported to EndNote version 8 reference managers to remove duplicates. Studies were separately evaluated for eligibility based on predetermined study selection criteria by two authors (F.W. and A.A.). Titles, abstracts, and keywords were initially screened and a full text of all potentially relevant articles were obtained for further assessment.

Two reviewers, T.B. and H.T., independently screened the titles and abstracts of all relevant records and assessed full texts for eligibility. Any disagreements were resolved between reviewers through discussion, and if no consensus was reached, a third reviewer (Z.A) was consulted. Additionally, a discussion with a senior author (A.B) used to resolve ant remaining disputes.

### Inclusion and exclusion criteria

In this systematic review and meta-analysis, we included studies that reported the proportion of knowledge, attitude or associated factors related to basic life support (BLS) among healthcare professionals in Ethiopia. For the purpose of this healthcare professionals defined as trained and licensed clinical personnel includes nurses, doctors, graduate medical student, medical students, internes, and residents. Studies reporting only the mean or median knowledge or attitude toward basic life support (BLS) and those did not report the proportion, were not included in the analysis. The review focusses solely on the study population without restriction on intervention, comparison, or outcomes. Only studies published in English were included. Additionally, studies without abstracts or full texts, anonymous reports, and qualitative research were excluded from the analysis.

### Data quality assessment

Two authors (A.A. and A.G) independently assessed the quality of each included study using the Joanna Briggs Institute (JBI) quality appraisal checklist. Any disagreement was resolved through discussion with a senior reviewer (B.B). Studies that scored 4 and above for all designs (cross-sectional and cohort studies), considered as low risk of bias, and considered as good quality.^25^ Studies scoring 3 and below were regarded as high-risk of bias or poor quality and excluded from analysis. All studies included in this systematic review met the criteria of good quality.

### Data extraction

This systematic review and meta-analysis primarily focus on the analysis of the prevalence of knowledge and attitudes related to BLS among healthcare professionals, with the secondary aim to identify factors that predict knowledge of BLS. Data were extracted by two independent authors (Z.A. and H.T.) using a pre-specified data collection Microsoft Excel spreadsheet, which was then monitored by two others (A.G. and A.A.). For each literature name of author, year of publication, study design, sample size, number of studied populations, odds ratio, confidence interval, study region, proportion of knowledge, and proportion of attitude were extracted. When data points were unclear or missing, the study's first and last authors were emailed to collect the information; if they did not respond, another email was sent a month later.

### Statistical analysis

The weighted prevalence of knowledge and attitude was calculated using the random effect model described by Desrimonian and Liard^26^ with a 95 % confidence interval (CI). The Meta and Forest Plot programs in STATA version 17 were used to perform statistical pooling and quantitative data synthesis. The I^2^ test, which estimates the proportion of total variation across studies, was used to assess heterogeneity in groups. An I^2^ value of more than 50 % indicates that the pooled data are heterogeneous. To address heterogeneity, subgroup analysis was performed on studies defining study regions. In addition, to assess a contribution of each individual study to the pooled prevalence of knowledge and attitude towards BLS, the sensitivity analysis was conducted. The presence of potential publication bias was evaluated by examining the symmetry of the funnel plot and conducted the meta-bias Egger test and meta-trim fill analysis.

## Results

**Fig. 1:**
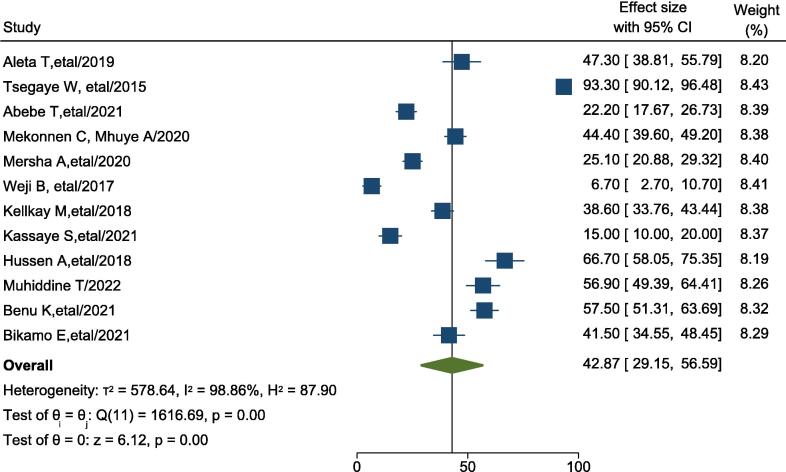
PRISMA 2020 flow diagram illustrating the study selection process from initial database searches to final inclusion.^27^***Note: n denotes the number of records at each stage of the selection process***.

A total of 2,527 studies were identified through database searches, registrations, and other sources. After removing duplicates, 241 records proceeded to title screening, resulting in the exclusion of 198 records. Of 43 abstracts reviewed, 6 of the studies were excluded. Subsequently, 37 full-text articles were reviewed for eligibility, of which 25 studies were excluded due to differences in study setting or outcomes. Finally, 12 studies fulfilled the inclusion criteria and were included in the final analysis (Fig. 1).

### Study characteristics

In this systematic review and meta-analysis 12 cross-sectional studies^4^, ^28^, ^29^, ^30^, ^31^, ^32^, ^33^, ^34^, ^35^, ^36^, ^37^ were included to estimate the pooled prevalence of knowledge among healthcare professionals on BLS. Out of 12 studies, 10 studies had reported on attitude on BLS among healthcare professionals. Regarding studied region four studies were from Amhara region,^4^, ^32^, ^33^, ^34^ five studies from Addis Ababa,^28^, ^29^, ^30^, ^31^ and three studies were from Debub,^35^ Tigray^36^ and Oromia region.^37^ In these studies, 3045 healthcare professionals were included (Table 1).

**Table 1:** Baseline characteristics of included studies.

S.N	Authors	Year	Region	Study design	Sample size	Prevalence of good knowledge	Prevalence of favorable attitude
1.	Aleta et al.^38^	2019	Addis Ababa	Cross-sectional	140	47.30 %	56.50 %
2.	Tsegaye et al.^37^	2015	Oromia	Cross-sectional	243	93.30 %	94 %
3.	Abebe et al.^32^	2021	Amhara	Cross-sectional	352	22.20 %	35.80 %
4.	Mersha et al.^33^	2020	Amhara	Cross-sectional	424	25.10 %	60.80 %
5.	Weji et al.^31^	2017	Addis Ababa	Cross-sectional	156	6.70 %	60 %
6.	Kellkay et al.^4^	2018	Amhara	Cross-sectional	397	38.60 %	−--
7.	Gebremehdin et al.^34^	2014	Amhara	Cross-sectional	461		42.90 %
8.	Kassaye et al.^30^	2021	Addis Ababa	Cross-sectional	196	15 %	63.20 %
9.	Hussen et al.^29^	2018	Addis Ababa	Cross-sectional	190	66.70 %	50 %
10.	Muhiddine^35^	2022	Debub	Cross-sectional	167	56.90 %	94 %
11.	Benu et al.^36^	2021	Tigray	Cross-sectional	245	57.50 %	−---
12.	Bikamo et al.^28^	2021	Addis Ababa	Cross-sectional	200	41.50 %	62.20 %

### Knowledge of healthcare professionals on basic life support (BLS)

**Fig. 2:**
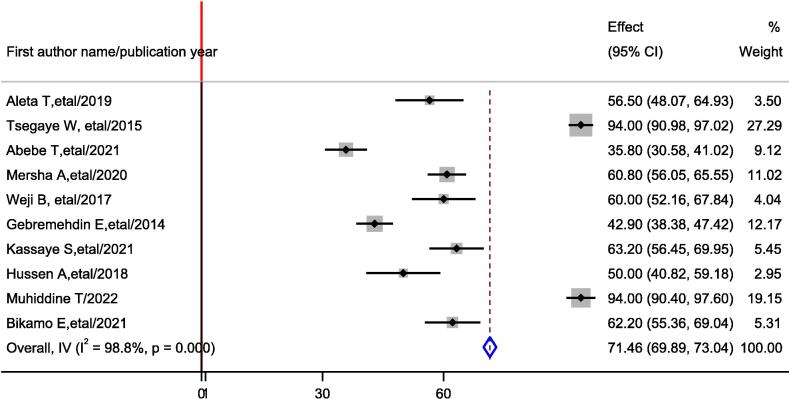
Forest plot showing the pooled prevalence of good knowledge of healthcare professionals on Basic life support in Ethiopia.

The prevalence of good knowledge towards Basic life support among healthcare professionals in Ethiopia was ranges from 6.7 % to 93.3 %. The random-effects model analysis from these studies revealed that, the pooled prevalence of good knowledge among healthcare professionals in Ethiopia was 42.87 % (95 % CI: 29.15–56.59 %; I^2^ = 98.86 %; p < 0.001) (Fig. 2).

Subgroup analysis was done through stratified by study region. Based on this the prevalence of good knowledge on BLS was 32.54 % (22.11 %, 42.96 %) in Amhara region, 56.9 % (49.39 %, 64.41 %) Debub region (Supplementary Fig. 1).

Publication bias: visual inspection of Meta funnel plot showed asymmetrical distribution (Supplementary Fig. 2). The egger regression value was test reviled that p = 0.42 which is suggestive of no publication bias (Supplementary Fig. 3).

### Attitude of healthcare professionals towards basic life support

**Fig. 3:**
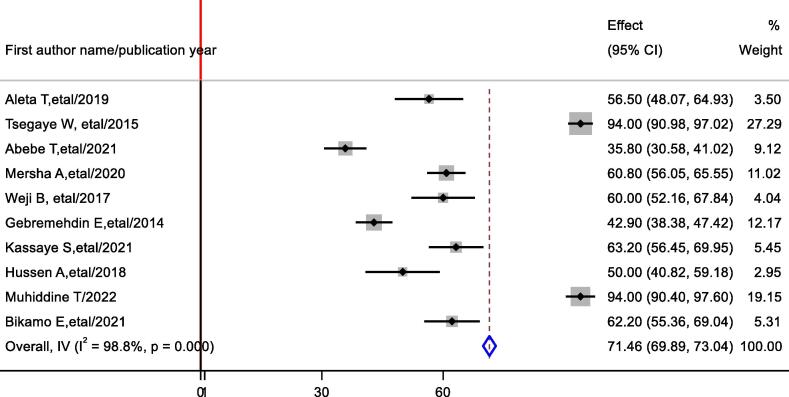
Forest plot showing the pooled prevalence favorable attitude of healthcare professionals on Basic life support in Ethiopia.

The prevalence of favorable attitude towards basic life support among healthcare professionals in Ethiopia was ranges from 35.8 % to 94 %. The random-effects model analysis from those studies revealed that, the pooled prevalence of favorable attitude among healthcare professionals in Ethiopia in was 71.46 % (95 % CI: 69.89 %, 73.04 %: I^2^ = 98.80 %; p < 0.0001) (Fig. 3).

Subgroup analysis was done through stratified by study region. Based on this the prevalence of favorable attitude on BLS was 59.05 % (54.70 %, 63.40 %) in Addis Ababa, 46.52 % (32.16 %, 60.89 %) in Amhara region (Supplementary Fig. 4).

Publication bias: Visual inspection of meta funnel plot showed asymmetrical distribution (Supplementary Fig. 5). The egger regression test value was p = 0.114 which is suggestive of no publication bias (Supplementary Fig. 6).

### Factors associated with knowledge towards BLS

Various studies reported several factors associated with knowledge of basic life support among healthcare professionals in Ethiopia. These include gender,^29^, ^36^ training on BLS,^28^, ^30^, ^33^, ^36^ previous exposure to situations requiring BLS,^4^, ^35^, ^38^ educational level,^4^, ^28^, ^38^ work experiences,^4^, ^33^, ^36^ working more than one hospital,^33^ and work unit.^4^ Similarly, clinical experiences,^29^ gender, training on BLS,^30^, ^33^ and adherence to CPR guidelines^33^ were reported as factors associated with attitude towards BLS.

Among those variables, seven variables were included in the pooled analysis across 12 studies. In this analysis, gender, previous training on BLS, and previous exposure to BLS were pooled. The pooled estimate showed that gender and previous exposure to BLS were significant predictors of knowledge about basic life support. Male healthcare professionals were 69 % less likely to have good knowledge of basic life support compared to female healthcare professionals (OR = 0.31;95 % CI: 0.07, 0.56) (Supplementary Fig. 7). Healthcare professionals with previous exposure to basic life support were 1.7 times more likely to have good knowledge of basic life support (OR = 1.74;95 % CI: 1.38, 2.11) (Supplementary Fig. 8).

## Discussion

Effective management of cardiac arrest requires prompt interventions supported by based on adequate knowledge and practical resuscitation skills.^39^ The quality of these intervention is crucial for improving patient survival and long term outcomes.^40^, ^41^ According to the American Redcross and other CPR/AED guidelines, survival decreased approximately by 7–10 % decrease for every minute that defibrillation is delayed.^42^, ^43^

This systematic review and meta-analysis estimate the pooled prevalence of good knowledge among healthcare professionals in Ethiopia. Accordingly, the pooled prevalence of good knowledge related to BLS among healthcare professionals in Ethiopia was 42.87 % (95 % CI: 29.15–56.59 %). The finding is comparable with the study conducted in Botswana 55.09 %^44^ and Pakistan 41.7 %.^45^ These similarities across settings might reflect the difficulties faced by healthcare professionals in low- and middle-income countries, such as limited access to regular BLS training, a lack of practical exposure, and the unavailability of BLS guidelines in their respective hospitals. These factors likely contribute to the constancy level of knowledge observed in these studies.

The findings of this systematic review and meta-analysis are lower than those reported in Nepal^46^ and Palestine.^47^ In Nepal, 67 % of healthcare professionals have moderate to good knowledge about basic life support.^46^ These differences may be explained by the purposive sampling technique used in the Nepal study, which may have led to inclusion of healthcare professionals with higher baseline knowledge of Basic life support. Additionally, a smaller sample size (n = 95) and greater access to BLS training may contribute to the higher knowledge level.^46^

In this study, the level of knowledge is higher than that reported in Egypt, 33.92 %.^48^ The difference might be attributed to variation in the operational definition used for the cut point used to classify participants as knowledgeable. The study conducted in Egypt uses cut point of 75 % to say knowledgeable, whereas the study conducted here in Ethiopia uses 50 %. The lower cut-off point used in the current study may account for a higher proportion of participants as knowledgeable.

In general, the prevalence of good knowledge towards basic life support among healthcare professionals in Ethiopian remains low, with only 42.8 % met the minimum standard, indicating a need for enhanced training and educational intervention.

In this systematic review and meta-analysis, the pooled prevalence of a favorable attitude in Ethiopia was 71.46 % (95 % CI: 69.89 %, 73.04 %), which is higher than that reported in Pakistan, 63 %,^49^ India, 59.9 %,^50^ and Saud Arabia 65.5 %.^51^ However, it is lower than the finding reported in Egypt (91 %),^52^ and Pakistan 91 %.^18^ The possible explanation for these differences might be due to differences in the study population. For example, the study in Egypt, included medical doctors who have greater exposure to BLS and ACLS. In our study, the participants include medical doctors, nurses, interns, anesthesiologist and graduate medical students, which may contribute for lower pooled prevalence.

In the subgroup analysis, variation with in the prevalence of favorable attitude were observed across study region. The prevalence of attitude was 59.05 % (54.70 %, 63.40 %) in Amhara region and 46.52 % (32.16 %, 60.89 %) in Addis Ababa.

The finding of this systematic review and meta-analysis reported that female healthcare professionals was knowledgeable than male healthcare professionals, which is supported by the study conducted in Addis Ababa Ethiopia,^29^ and Saudi Arabia.^53^ Evidence from previous studies suggests reasons why female healthcare professionals tend to have higher BLS knowledge than male healthcare professionals. The first experimental study found that females are more engaged in BLS training than males, indicating stronger educational engagement.^54^

Healthcare professionals who had previous exposure on situations requiring BLS were 1.74 times more likely to have adequate knowledge of basic life support compared to those without exposure. This finding is consistent with the studies conducted in Addis Ababa,^38^ Gondar,^4^ and Debub, Ethiopia.^35^ A possible explanation is that professionals with previous BLS exposure are motivated to learn about lifesaving interventions, leading them to engage with BLS guidelines, and training materials. Moreover, hands-on practical experiences and real-life exposure enhance the acquisition of knowledge and skill.

The finding of this study has important implications for policymakers, stakeholders, and other concerned bodies. They can be developing strategies aimed at improving the knowledge, and attitude of healthcare professionals towards basic life support. These include promoting continuous in-services training, accessing updated BLS guidelines, and facilitating simulations for all healthcare professionals.

### Limitations

The result of this systematic review and meta-analysis can serve as a basis for drawing conclusions by minimizing inconsistences across the studies. However, due to insufficient number of primary studies it is difficult to pooled factors associated with attitudes of healthcare professionals towards BLS. This systematic review and meta-analysis were not registered in the PROSPRO registry. Additionally, only studies published in English were included: relevant studies published in local or other language may have been missed, introducing potential language bias.

## Conclusion

The overall prevalence good knowledge among healthcare professionals in Ethiopia remains below the Minimum standards, while their attitude towards basic life support was suboptimal. Male healthcare professionals less likely to have adequate knowledge on basic life support, where as those with pervious exposure to basic life support demonstrate good knowledge.

## Funding information

None to declare.

## Data availability

All data used in this systematic review and meta-analysis are openly available in Zendo under a CC0 1.0 Universal license. The data set includes full data extraction sheet for with study characteristics, sample sizes, outcomes, and other variables used for pooling estimates on Basic life support competency among healthcare professionals in Ethiopia: A systematic review and meta-analysis. The data extraction dataset can be accessed at https://doi.org/10.5281/zenodo.17006580 or https://zenodo.org/records/17006580.

## CRediT authorship contribution statement

**Gebremeskel Kibret Abebe:** Writing – review & editing, Writing – original draft, Supervision, Formal analysis, Data curation, Conceptualization. **Addis Wondmagegn Alamaw:** Visualization, Validation, Supervision, Data curation, Conceptualization. **Biruk Beletew Abate:** Visualization, Validation, Supervision, Software, Resources, Methodology, Conceptualization. **Befkad Derese Tilahun:** Formal analysis, Data curation, Conceptualization. **Alemu Birara Zemariam:** Visualization, Validation, Supervision. **Tesfaye Engdaw Habtie:** Visualization, Software, Methodology, Investigation, Conceptualization. **Fekadu Takele Wolie:** Supervision, Software, Data curation.

## Declaration of competing interest

The authors declare that they have no known competing financial interests or personal relationships that could have appeared to influence the work reported in this paper.
