# Supplementary material for: Basic life support competency among healthcare professionals in Ethiopia: a systematic review and meta-analysis, 2025
Source: Resusc Plus. 2025 Sep 11;26:101098. doi: 10.1016/j.resplu.2025.101098 (PMC12492288; doi:10.1016/j.resplu.2025.101098)
Supplement: Supplementary Data 1 [file mmc1.docx]

Supplementary figure 1:forest plot showing subgroup analysis knowledge of healthcare professionals on basic life support by study region.
