# Supplementary material for: Basic life support competency among healthcare professionals in Ethiopia: a systematic review and meta-analysis, 2025
Source: Resusc Plus. 2025 Sep 11;26:101098. doi: 10.1016/j.resplu.2025.101098 (PMC12492288; doi:10.1016/j.resplu.2025.101098)
Supplement: Supplementary Data 6 [file mmc6.docx]

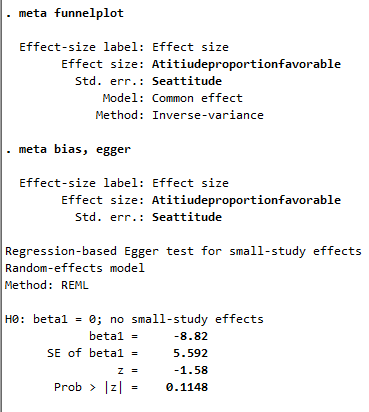


Supplementary figure 6: Meta bias, egger test showering publication bias on attitude of healthcare professionals regarding basic life support.
