# Supplementary material for: Basic life support competency among healthcare professionals in Ethiopia: a systematic review and meta-analysis, 2025
Source: Resusc Plus. 2025 Sep 11;26:101098. doi: 10.1016/j.resplu.2025.101098 (PMC12492288; doi:10.1016/j.resplu.2025.101098)
Supplement: Supplementary Data 8 [file mmc8.docx]

Supplementary figure 8: Forest plot showing association of pervious exposure and knowledge of BLS among healthcare professionals in Ethiopia.
